# Ex-vivo limb perfusion in military and civilian medicine: inspired by ex-vivo organ perfusion, pioneered for traumatic limb amputation and peripheral nerve regeneration

**DOI:** 10.1186/s40779-025-00656-6

**Published:** 2025-10-29

**Authors:** Kirsten Haastert-Talini, Katherina Katsirntaki, Svenja Kankowski, Alexander Kaltenborn, Falk von Lübken, Christine Falk, Christopher Werlein, Danny Jonigk, Arjang Ruhparwar, Bettina Wiegmann

**Affiliations:** 1 Hannover Medical School, Institute of Neuroanatomy and Cell Biology, 30625 Hannover, Germany; 2 Center for Systems Neuroscience (ZSN), 30625 Hannover, Germany; 3 Department for Cardiothoracic, Transplantation and Vascular Surgery, Hannover Medical School, 30625 Hannover, Germany; 4 Lower Saxony Center for Biomedical Engineering, Implant Research and Development (NIFE), 30625 Hannover, Germany; 5 Department of Trauma and Orthopedic Surgery, Plastic, Hand and Reconstructive Surgery, Armed Forces Hospital Westerstede, 26655 Westerstede, Germany; 6 Hannover Medical School, Institute of Transplant Immunology, 30625 Hannover, Germany; 7 German Center for Lung Research (DZL), BREATH Hannover, 30625 Hannover, Germany; 8 Aachen University Medical Center, Institute of Pathology, RWTH Aachen University, 52074 Aachen, Germany; 9 Hannover Medical School, Institute of Pathology, 30625 Hannover, Germany

**Keywords:** Ex vivo perfusion, Ex-situ perfusion, Ex vivo limb perfusion, Traumatic limb amputation, Nerve regeneration, Autologous replantation, Wallerian degeneration, Peripheral nerve regeneration

## Abstract

**Background:**

Traumatic amputations have increased worldwide over the past two decades and are expected to increase by 72% by 2050. Surgical replantation provides superior functional recovery and patient satisfaction but is limited to specialized centers and restricted by short ischemia times, due to life-over-limb prioritization in patient care. To overcome these limitations, we developed an ex vivo limb perfusion system (EVEP) to extend limb viability and, for the first time, investigate its impact on peripheral nerve regeneration, a key prerequisite for functional recovery following replantation.

**Methods:**

Hind limbs of 6 healthy pigs were amputated, and after 2 h of warm ischemia, limbs were either perfused normothermally for 6 h with PerfadexPlus® ± medication using in-house developed EVEP or stored statically (4 °C vs. room temperature). Perfusion parameters, blood gas analysis, serum markers, cytokine levels, thermal imaging, colloid oncotic pressure, weight gain, joint mobility, peripheral nerve histomorphometric and stereological analyses were performed.

**Results:**

Data confirm a valid and reliable EVEP with an optimized perfusion protocol. Comparison of perfusion groups revealed lower serum injury markers in the medication group, which included methylprednisolone treatment. Additionally, the medication group exhibited reduced weight gain and preserved unrestricted joint mobility, but concurrently led to a significant decrease in pro-regenerative cytokine levels associated with Wallerian degeneration (WD).

**Conclusions:**

In general, EVEP mitigates ischemia-related damage and facilitates ex vivo induction of WD, a critical prerequisite for nerve regeneration, functional recovery, and prevention of neuroma formation with subsequent phantom pain, by establishing the pro-regenerative environment for WD, which is further amplified by omitting the anti-inflammatory methylprednisolone.

**Supplementary Information:**

The online version contains supplementary material available at 10.1186/s40779-025-00656-6.

## Background

Over the last 20 years, there has been a significant increase in the incidence and prevalence of traumatic amputations worldwide, both as a result of accidents at work or during leisure time, and terrorist attacks on civilians and amputations in military war operations [1]. Traumatic amputations worldwide are projected to increase by 72% by 20,250 [2–4]. This is particularly due to the significant increase in violent conflicts and harmful activities affecting both civilian and military populations, as well as considerable advances in military protective equipment, which reduce the mortality rate while increasing the number of survivors with traumatic amputations. This has far-reaching socioeconomic consequences for those affected, leading in turn to an increased risk of developing psychiatric disorders (e.g., depression) [5–8]. Therapeutic options of prosthetic and surgical treatment were compared in the civilian sector by the Lower Extremity Assessment Project and in the military sector by the Military Extremity Trauma Amputation/Limb Salvage study, with surgical replantation resulting in significantly better functionality and patient satisfaction [9, 10]. However, successful implementation requires a logistical masterpiece in a specialized hospital with a well-coordinated interdisciplinary team, with stabilizing and saving patients’ lives taking precedence over preserving limbs. An additional challenge is that ischemia time of muscle-rich limbs in standard static cold storage is only 4–6 h and can quickly be exceeded in this environment [9], especially in military combat missions (e.g., tactical evacuation), where amputation is therefore the current treatment of choice. Only 0.4% of patients with traumatic transfemoral amputations currently receive replantation [7].

Ex vivo perfusion is a suitable approach to effectively meet these challenges in the future. Over the last decade, ex vivo perfusion has become a well-established alternative to static cold storage in organ transplantation, as it uses temperature-controlled perfusion with blood-like, oxygen-rich solution to create an almost physiological environment for organs [11]. This significantly reduces the cold ischemic time and thus also the harmful ischemia–reperfusion injury (IRI), resulting in better outcomes after transplantation [12, 13]. Additionally, effective and extended organ preservation allows for greater geographical flexibility in donor management, as well as targeted therapy and re-evaluation of marginal donor organs with conversion from non-transplantable to transplantable ones [14, 15], thereby significantly expanding the donor pool while reducing the growing gap between potential organ donors and recipients. These breakthroughs inspired the evolution of other highly innovative ex vivo applications, such as regenerative approaches and immunomodulation [16, 17]. However, a corresponding system for ex vivo limb perfusion (EVEP), which offers significant advantages for the care of patients with traumatic amputations, does not yet exist. Current state of knowledge on these developments and limb-specific perfusions protocols is limited and heterogeneous [18–21], and no existing protocol address the key uniqueness of EVEP, the Wallerian degeneration (WD) within the axotomized peripheral nerves, which is indispensable for peripheral nerve regeneration and paves the way for successful functional recovery after limb replantation while avoiding pathological neuroma formation associated with phantom limb pain development [22–24]. Based on our experience with ex vivo perfusion of solid organs, the study aims to establish a valid EVEP platform with a reproducible protocol and dedicated limb-specific analytical methods, designed to explore the potential of EVEP to support peripheral nerve regeneration in ex vivo-perfused limbs.

## Methods

### Ethical approval, limb procurement, and experimental groups

All animal protocols were performed in compliance with the German Animal Welfare Law and the European Communities Council´s directive (Directive 2010/63/EU) for the protection of animals for experimentation. Experiments were carried out after approval by the local animal experimentation ethics committee and the animal welfare officer of Hannover Medical School (MHH-§4-2019/247).

For limb procurement, 6 healthy female domestic pigs [(38.4 ± 1.5) kg] were administered 1 ml/10 kg body weight tiletamine zolazepam (Zoletil 100, Vibrac AG, Switzerland) and 1 ml atropine sulphate (Atropine Sulfate 0.5 mg/ml, B. Braun, Germany) intramuscularly, also 20 ml narcofol (Narcofol 10 mg/ml, Cp-Pharma, Germany) intravenously before receiving 20 ml release (Release 300 mg/ml, pentobarbital sodium, WDT, Germany) for euthanasia. After sterile washing, animals were exsanguinated via sternotomy with cardiac apex resection. Via median laparotomy, the abdominal aorta and inferior vena cava were cannulated below the inferior mesenteric vessels and perfused antegrade with 1000 ml of sodium chloride (NaCl) 0.9% and 5000 U heparin sodium to flush out residual blood, followed by cannulation of the left and right iliac artery and vein.

For final amputation of the hind limbs, median laparotomy was continued towards the tail, dissecting muscle and connective tissue to allow disarticulation of the hip joint. After 2 h of warm ischemia at room temperature (RT), simulating the worst-case scenario of traumatic amputation with inadequate storage, amputated hind limbs were preserved for 6 h. EVEP group was normothermically perfused using acellular PerfadexPlus®, with drugs added to the perfusate in one half of the group (w group; *n* = 3) according to the ex-vivo lung perfusion protocol (1 ampoule 10 ml Frekavit-Multivitamin, 20 U human insulin, 4.5 g piperacillin/tazobactam-azobactam, and 500 mg methylprednisolone-21-hydrogen succinate) and additionally treated intra-arterially with 6.5 µg/h of the vasodilator alprostadil [12, 13], while the other half was perfused without medication (w/o group; *n* = 3). Control limbs were stored statically on ice inside a 4 °C storage room (CS group; *n* = 3) or at RT (RT group; *n* = 3).

### Limb preservation with ex vivo limb perfusion

**Fig. 1:**
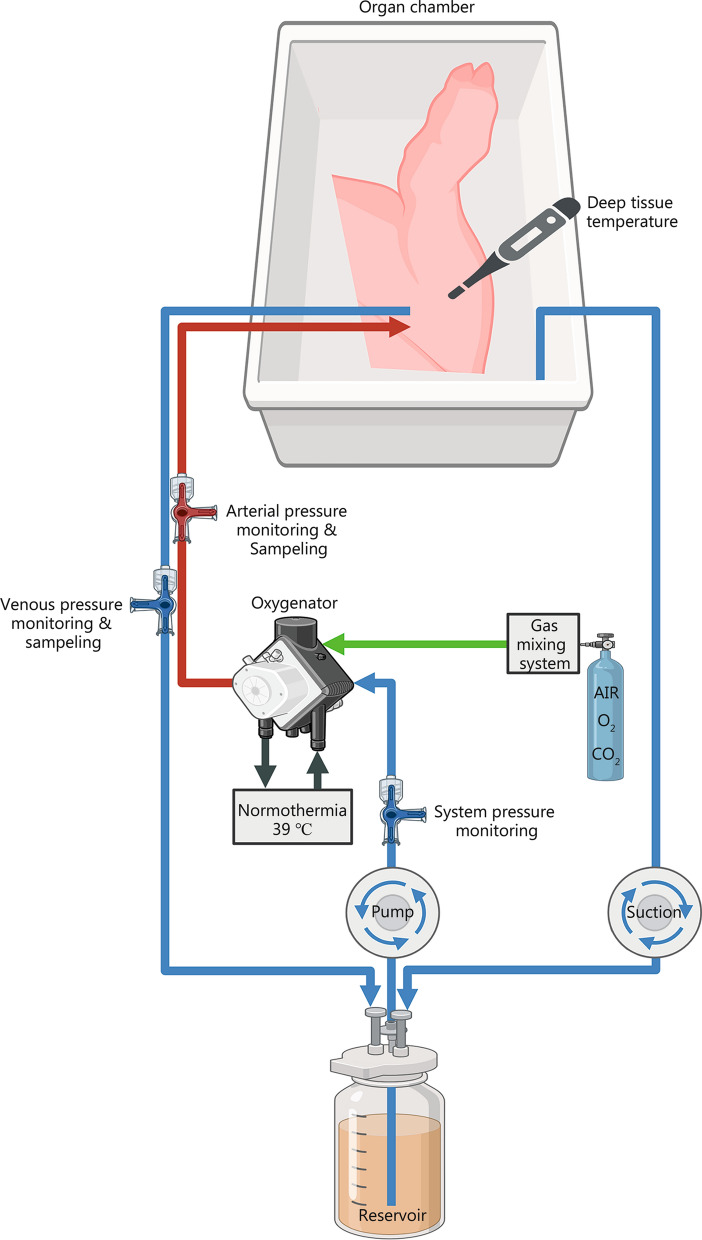
Experimental setup of ex vivo perfusion. The porcine hind limb was cannulated via the respective common iliac arteries and veins, and monitoring and sampling were performed via three-way stopcocks. Starting from the reservoir, an oxygenator with an integrated heat exchanger was introduced into the arterial line. The perfusate was oxygenated and decarboxylated by this oxygenator, kept at a constant temperature of 39 °C, and pumped the perfusate into the limb by a double roller pump

Six-hour EVEP was performed with a customized heart–lung machine (SIII system, Stöckert Instrumente GmbH, Germany) with a double roller pump, an oxygenator, an electronic gas regulator, and a reservoir. After priming the system with 10 ml tris(hydroxymethyl)aminomethane (TRIS), 5 ml 40% glucose and 1 L PerfadexPlus®, the integrated heat exchanger kept the perfusate at porcine normothermic temperature of 39 °C, while pulsatile, antegrade perfusion was performed at 0.1 ml/(min·kg) limb weight (Fig. 1). Physiological pH and glucose levels were maintained by titrated addition of TRIS and 40% glucose, respectively. Oxygen (O_2_) and carbon dioxide (CO_2_) supply was maintained by a gas flow of 1.1 L/min with 0.3 L/min O_2_ and 0.1 ml/min CO_2_ at clinically relevant partial pressures (pO_2_ 150–250 mmHg, pCO_2_ 35–48 mmHg). As soon as the reservoir reached < 50 ml volume, PerfadexPlus® was added up to 200 ml. Control limbs were flushed with 250 ml NaCl 0.9% and 1250 U heparin sulfate to wash out any residual blood before being statically stored.

### Analyses under corresponding storage

For comparative analysis, in the EVEP groups, hourly systemic pressure (SP), mean arterial pressure (MAP), and venous pressure (MVP) were measured, from which systemic vascular resistance (VR) was calculated. Temperature control was assessed by system temperature (ST) generated by the heat exchanger, the mean limb surface temperature recorded with the thermal imaging camera (VarioCam®HDx research 645S, Germany), and the deep intramuscular temperature measured with a thermometer. In addition to colloidal oncotic pressure (COP), perfusate analysis included arterial and venous blood gas analysis (BGA), measuring pH, pO_2_, pCO_2_, sodium, potassium, lactate, and glucose, and allowed the calculation of the glucose uptake rate (GUR) [GUR = (glucose_arterial_ — glucose_venous_)/(glucose_arterial_ × 100%)]. Lactate dehydrogenase (LDH), creatine kinase (CK), myoglobin, and cytokines [e. g. interleukins (IL)-1a, IL-6] were analyzed every 2 h. Limb weight and mobility in the knee and ankle joints were measured before and after perfusion, with histological sampling of the pectineus muscle, cutis, and subcutaneous fatty tissue performed at the same time. Time points for blood sampling (BGA, serum markers, cytokines), COPs, and temperature measurements on the surface and at depth were the same for the control groups.

### Histomorphometric and stereological nerve analysis

Samples were taken immediately after euthanasia and at the end of each experiment. The sciatic nerve was exposed along its entire length from the amputation site to the heel. After identifying the proximal sciatic nerve muscle branch (RMoSN) and the distal common fibular nerve (CFN), 5-mm samples were taken and fixed as previously described [25]. The myelin sheath was stained with 1% potassium dichromate, 25% ethanol, and hematoxylin, embedded in Epon, and cut into 1 µm cross sections. Following, staining was intensified with toluidine blue. Stereological nerve analysis was performed microscopically in two randomly selected sections per sample, using the two-dimensional dissection method, in which the total number of myelinated fibers was determined at 100 × magnification in cross-section areas and marked at 10 × magnification [26]. A 30 µm × 30 µm counting frame with a grid size of 125 µm × 125 µm (RMoSN) or 175 µm × 175 µm (CFN) was used to quantify the number and density of myelinated fibers, as well as the number and density of fibers with swollen myelin sheaths and impaired myelin sheath integrity. In addition, data were used to quantify nerve fiber density ratio by digitizing and analyzing the area ratio between the epineurium, the connective tissue surrounding the nerve, and the nerve fascicles, surrounded by the perineurium, from two cross sections per sample at 20 × magnification. Nerve morphometry analysis was performed at 100 × magnification on eight image fields per sample. Assuming that axons have a circular shape, analysis was performed using ImageJ software and the *g*-ratio plug-in to determine axon diameter, myelin layer thickness, and resulting fiber diameters. Finally, nerve fiber diameters (≤ 6 µm and > 6 µm) within the analyzed nerve segments were recorded [26, 27].

### Histopathologic assessment

Tissue samples from skin, soft tissue, and skeletal muscle were taken immediately after euthanasia, after 2 h of perfusion, and at the end of each experiment, fixed in 4% buffered formaldehyde, embedded in paraffin, cut into 2 µm thick slices, and stained with haematoxylin-eosin to assess edema and necrosis. Conventional microscopy was used to semi-quantitatively grade soft tissue edema and skeletal muscle necrosis from absent (0: no visible changes) to minimal (1: focal or minimal extension of soft tissue fibers/only single necrotic cells up to 5%) to moderate (2: generalized or larger extension of soft tissue fibers/5–10% visible necrotic cells). Representative images were acquired, and image processing was performed using ImageJ software.

### Statistical analysis

GraphPad Prism version 10.2.3 was used for statistical analysis, whereby for the normality test, the D’Agostino-Pearson test, the Shapiro-Wilk normality test, and the Kolmogorov-Smirnov test were used. Data was summarized using a time curve; each row represents a different time point, so that matching values were summarized in sub-columns for statistical analysis. Repeated measures (RM) two-way ANOVA and Tukey’s multiple comparison test were used to analyze grouped data over a time curve. A multiple-adjusted *P*-value was given for each comparison. Data were presented as mean ± standard deviation (SD). Nerve histomorphometrical data, weight gain, and joint mobility were analyzed for normal distribution and either subjected to a non-parametric Kruskal-Wallis test followed by Dunn’s multiple comparisons post-hoc test or to parametric one-way ANOVA followed by Tukey’s multiple comparisons post-hoc test. Histology samples were semi-quantitatively graded (0, 1, or 2) by an experienced pathologist, blinded to the experimental setup as described above, for each sample. Afterwards, mean and SD were calculated for each group. The different groups were compared for statistical differences via the Chi-square test. *P* < 0.05 indicates statistical significance.

## Results

### Ex vivo limb perfusion

#### Monitoring parameters, BGA, and histology confirm reliable EVEP

**Fig. 2:**
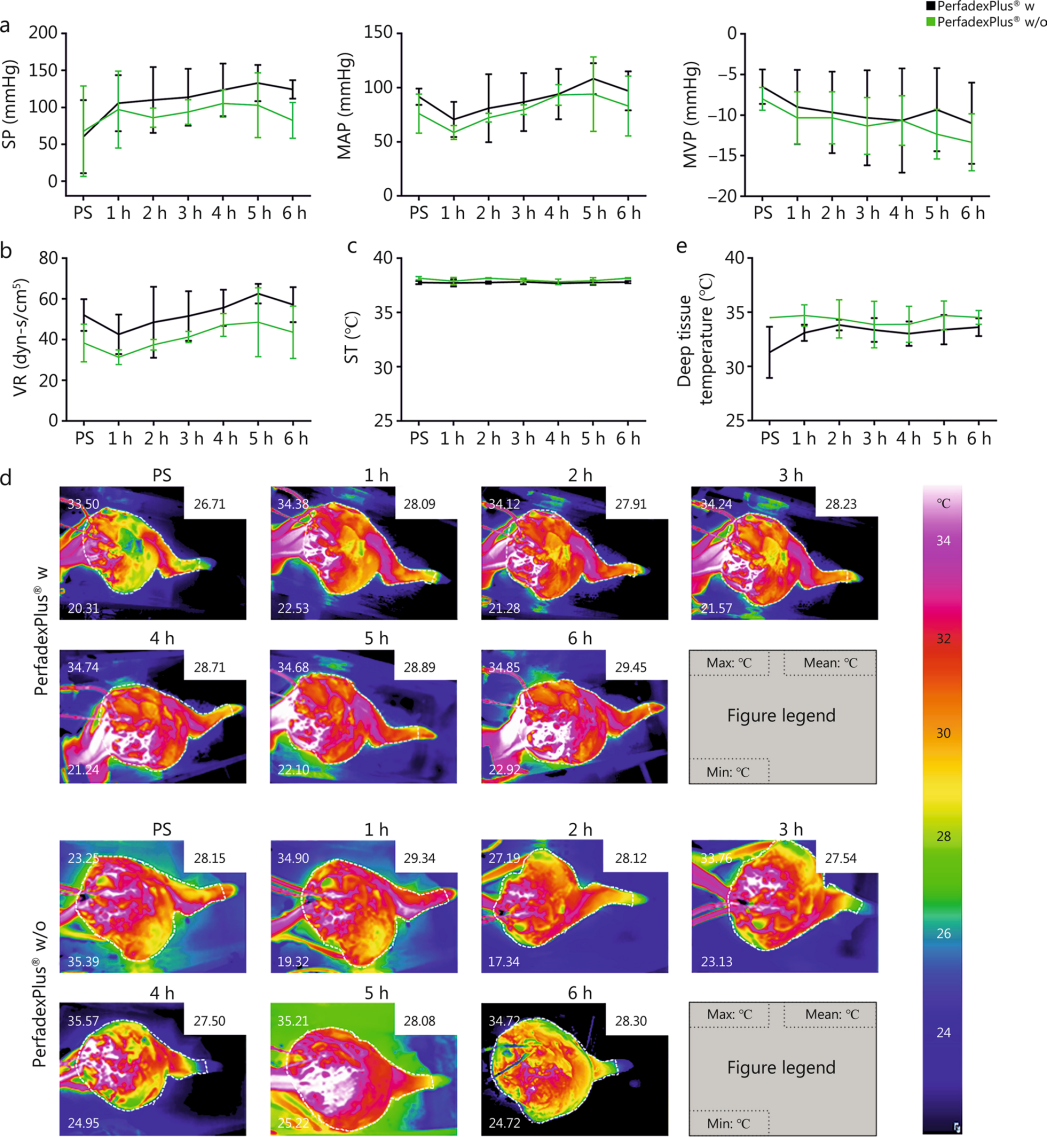
Ex vivo monitoring of perfused limbs over a 6-hour experimental time. **a** Systemic pressure (SP), Mean arterial pressure (MAP), Mean venous pressure (MVP). **b** Calculated vascular resistance (VR). **c** Systemic temperature (ST). **d** Examples of the surface temperatures in perfusion groups without medication and with medication. **e** Deep tissue temperature. For each figure, *n* = 3 was analyzed. Data of the perfusion group with (black) and without (green) medication were shown as mean values and standard deviations. Repeated measures two-way ANOVA and Tukey’s multiple comparison test were used to analyze grouped data over time. A multiple-adjusted *P*-value was given for each comparison. For Fig. **d**, the mean, maximum, and minimum temperatures were shown with the corresponding temperature color palette from 34 to 24 °C. PS perfusion start, w with medication, w/o without medication

**Fig. 3:**
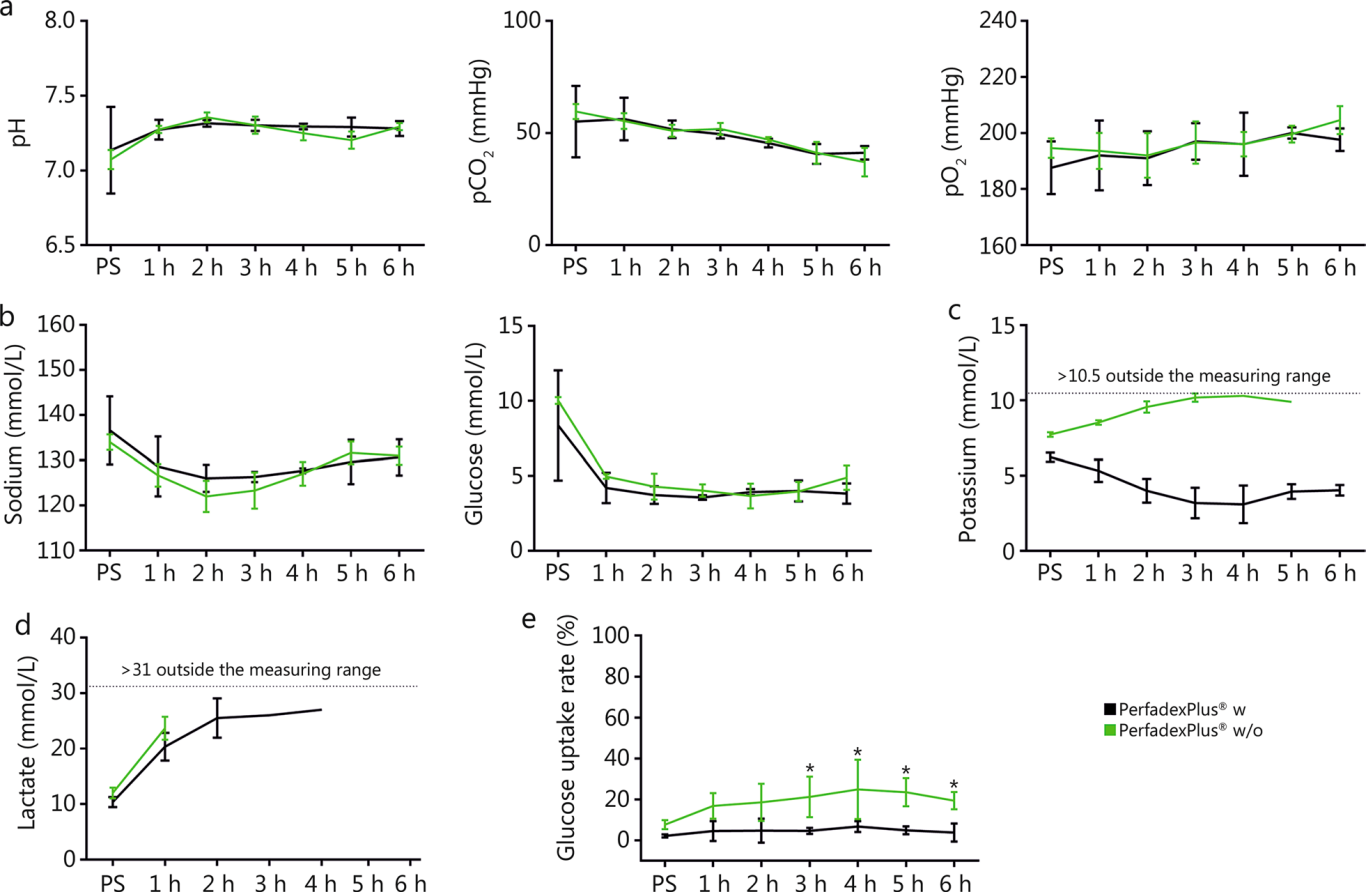
Blood gas analysis and metabolic activity of ex vivo perfused limbs. **a** pH, pCO_2_, pO_2_. **b** Sodium, Glucose. **c** Potassium. **d** Lactate. **e** Glucose uptake rates. For each figure, *n* = 3 was analyzed. Potassium and lactate could not be statistically evaluated as the values were out of range during the experiments. Glucose uptake rates were calculated from the available data (*n* = 3). Data of the perfusion group with (black) and without (green) medication were shown as mean values and standard deviations. Repeated measures two-way ANOVA and Tukey’s multiple comparison test were used to analyze grouped data over time. A multiple-adjusted *P*-value was given for each comparison. ^*^P < 0.05. CO_2_ carbon dioxide, O_2_ oxygen, PS perfusion start, w with medication, w/o without medication

SP [(110.0 ± 23.9) mmHg vs. (91.0 ± 13.1) mmHg], MAP [(90.0 ± 12.0) mmHg vs. (80.0 ± 2.3) mmHg] and MVP [(-10.0 ± 1.5) mmHg vs. (− 11.0 ± 1.7) mmHg] showed stable, comparable values during 6-hour EVEP for perfused limbs between w and w/o group, whereby VR of w group was constantly higher compared to w/o group [(53.0 ± 6.4) mmHg vs. (41.0 ± 6.0) mmHg] (Fig. 2a,b). Heat exchanger kept ST constant at (38.0 ± 0.1) °C (Fig. 2c), resulting in comparable, homogeneously distributed stable mean surface temperatures ranging from 26.7 to 29.5 °C (w) and 28.2 to 28.3 °C (w/o), which was already achieved in the first hour of perfusion (Fig. 2d). The same was true for deep tissue temperature, which was on average (33.1 ± 0.8) °C (w) and (34.4 ± 0.4) °C (w/o) (Fig. 2e). Both perfusion groups showed significant temperature increases from perfusion start (PS) to the end of perfusion by 3.4 °C (w) and 2.3 °C (w/o) respectively. However, there was an overall discrepancy between the ST and superficial temperature of about 10 °C and the deep temperature of about 6 °C. BGA showed no significant differences in pH, pCO_2_ and pO_2_ between the w and w/o groups (Fig. 3a). After 2 h of ischemia, PS acidic pH was (7.1 ± 0.3) (w) and (7.1 ± 0.1) (w/o), with pCO_2_ of (55.1 ± 16.0) mmHg (w) and (59.6 ± 3.3) mmHg (w/o), which stabilized to near physiological values within the first hour of perfusion [w: (7.3 ± 0.5) and (56.3 ± 9.5) mmHg, w/o: (7.3 ± 0.02) and (55.4 ± 3.6) mmHg], and remained constant with TRIS addition (w: 87.0 ml, w/o: 76.0 ml) and fine adjustment of CO_2_ gas flow. This also applies to pO_2_, which remained constant during 6-hour perfusion with an average of (194.5 ± 4.4) mmHg (w) and (196.8 ± 4.3) mmHg (w/o). Skeletal muscle necrosis, which is mainly caused by hypoxia and insufficient blood flow, was a rare event, negligible in both groups, with no statistically significant differences between the experimental conditions or time points of sampling (Additional file 1: Fig. S1 and Table S1).

#### Supplementary medication in perfusion solution supports metabolic activity

There were no significant differences in sodium and glucose, within the first hour of perfusion they stabilized to physiological values of (128.7 ± 6.7) mmol/L (w) and (126.0 ± 2.5) mmol/L (w/o) for sodium and (4.2 ± 1.0) mmol/L (w) and (4.9 ± 1.5) mmol/L (w/o) for glucose (Fig. 3b), which was kept constant by 40% glucose application (w: 24.3 ml, w/o: 15.6 ml). At PS, potassium levels in both groups were outside physiological references, with potassium in the w group starting at (6.2 ± 0.3) mmol/L, falling to a minimum of (3.2 ± 1.0) mmol/L in the fourth hour, and remaining stable at (4.1 ± 0.7) mmol/L until the end. In contrast, the w/o group showed an initial value of (8.8 ± 1.8) mmol/L, which rose steadily and was above the measurement range from the fifth hour of perfusion (Fig. 3c). This also applied to lactate, which was already pathologically elevated in both groups at PS with (10.4 ± 0.9) mmol/L (w) and (12.0 ± 1.0) mmol/L (w/o), and was above the measuring range from the second (w/o) and fourth hour onwards (w) (Fig. 3d). A statistical analysis was therefore not possible for either lactate or potassium. Mean GUR was (4.5 ± 1.4) % (w) and (18.9 ± 5.7) % (w/o), with GUR of the w/o group being significantly higher than that of the w group from the third hour onwards (Fig. 3e).

#### Drug additives lead to significantly lower serum markers and less weight gain, with better joint mobility

**Fig. 4:**
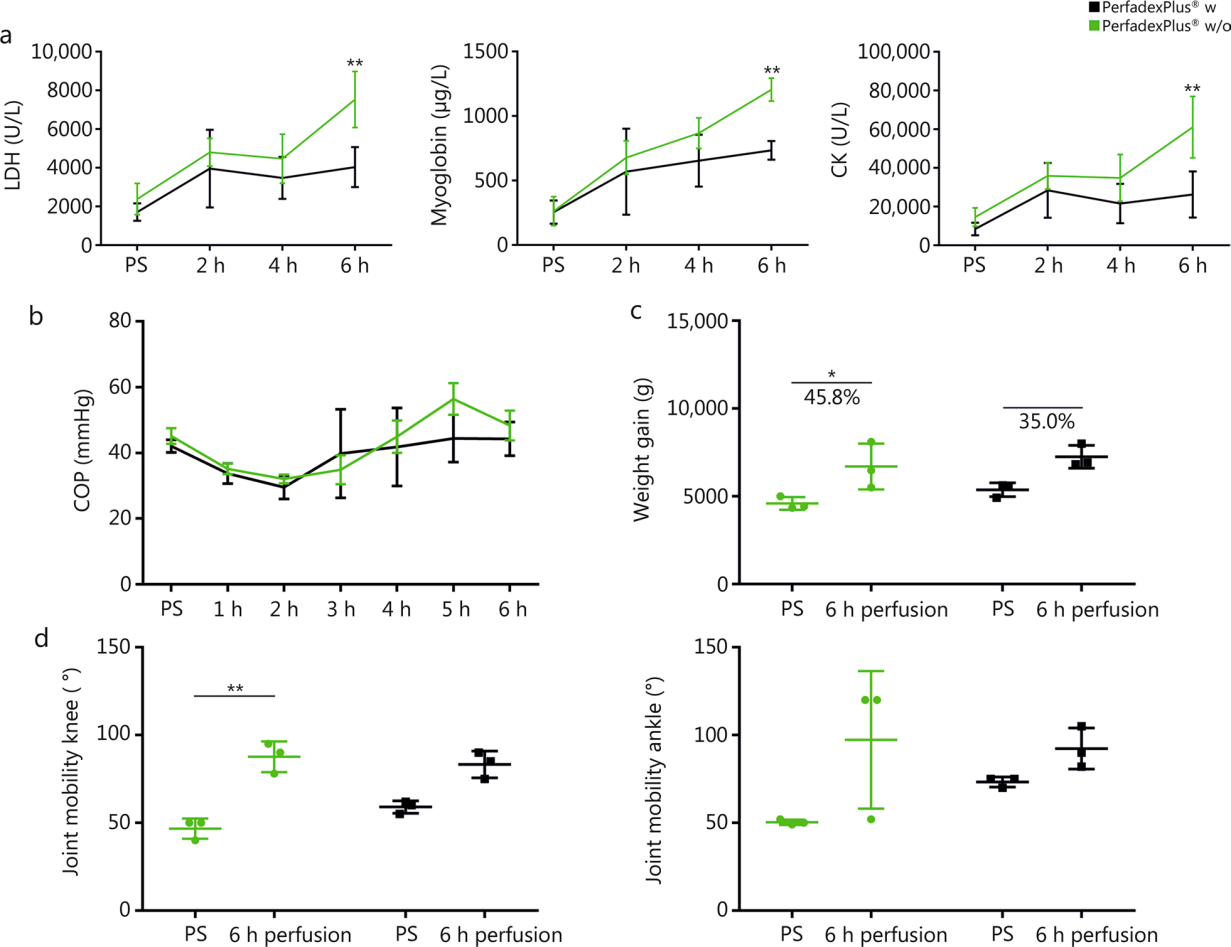
Serum markers and edema relevant aspects of ex vivo perfused limbs. **a** Lactate dehydrogenase (LDH), Myoglobin, Creatine kinase (CK). **b** Colloid oncotic pressure (COP). **c** Weight gain. **d** Joint mobility of the knee and ankle. For each figure, *n* = 3 was analyzed. Data of the perfusion group with (black) and without (green) medication were shown as mean values and standard deviations. Percentages stood for the weight gain between PS and 6 h perfusion. Joint mobility data (knee and ankle) were analyzed for normal distribution and either subjected to a non-parametric Kruskal-Wallis test followed by Dunn’s multiple comparisons post-hoc test or to parametric one-way ANOVA followed by Tukey’s multiple comparisons post-hoc test. Repeated measures two-way ANOVA and Tukey’s multiple comparison test were used to analyze grouped data over time. A multiple-adjusted *P*-value was given for each comparison. ^*^P < 0.05, ^**^P < 0.01. PS perfusion start, w with medication, w/o without medication

There was a time-dependent increase in LDH, myoglobin and CK in both EVEP groups, but this was significantly less pronounced in the w group, as shown by respective mean values [LDH: (3297.0 ± 1087.0) U/L vs. (4800.0 ± 2114.0) U/L; myoglobin: (553.5 ± 209.6) µg/L vs. (753.8 ± 392.7) µg/L; CK: (21,214.0 ± 8976.0) U/L vs. (36,627.0 ± 19,044.0) U/L; Fig. 4a] and their maximum at perfusion end [LDH: (4040.0 ± 2618.0) U/L vs. (7537.0 ± 1615.0) U/L; myoglobin: (734.0 ± 196.4) µg/L vs. (1205.0 ± 471.7) µg/L; CK: (26,302.0 ± 2435.0) U/L vs. (61,095.0 ± 24,235.0) U/L; Fig. 4a]. No significant differences in COP were observed during EVEP. Initial values [(42.1 ± 2.0) mmHg (w) vs. (45.2 ± 2.4) mmHg (w/o)] reached their minimum after 2 h [(29.6 ± 3.5) mmHg (w)] and 2.5 h [(32.1 ± 1.1) mmHg (w/o)], and after respective perfusate administration [(1957.0 ± 1098.0) ml (w) vs. (2525.0 ± 375.0) ml (w/o)] to maintain perfusion, COP was comparable at the end [(44.3 ± 5.2) mmHg (w) vs. (48.4 ± 4.5) mmHg (w/o)] (Fig. 4b). Both groups showed a weight gain, 35% from (5370.0 ± 789.4) g to (7252.0 ± 1299.6) g in the w group and significantly from (4593.3 ± 363.6) g to (6700.0 ± 1310.8) g, corresponding to 45.8% in the w/o group (Fig. 4c). Histological evaluations showed in both groups a time-dependent increase in edema, although it’s grading was significantly higher in the w/o group (Additional file 1: Fig. S1 and Table S1). This was reflected in a decrease in mobility in knee joints from (59.0 ± 3.6) ° to (83.3 ± 7.7) ° in the w group and significantly from (46.7 ± 5.7) ° to (87.7 ± 1.4) ° in the w/o group, and ankle joints from (73.3 ± 2.9) ° to (92.3 ± 11.7) ° in the w group and (50.3 ± 1.5) ° to (97.3 ± 78.5) ° in the w/o group (Fig. 4d).

#### Methylprednisolone significantly suppresses pro-inflammatory immune response

**Fig. 5:**
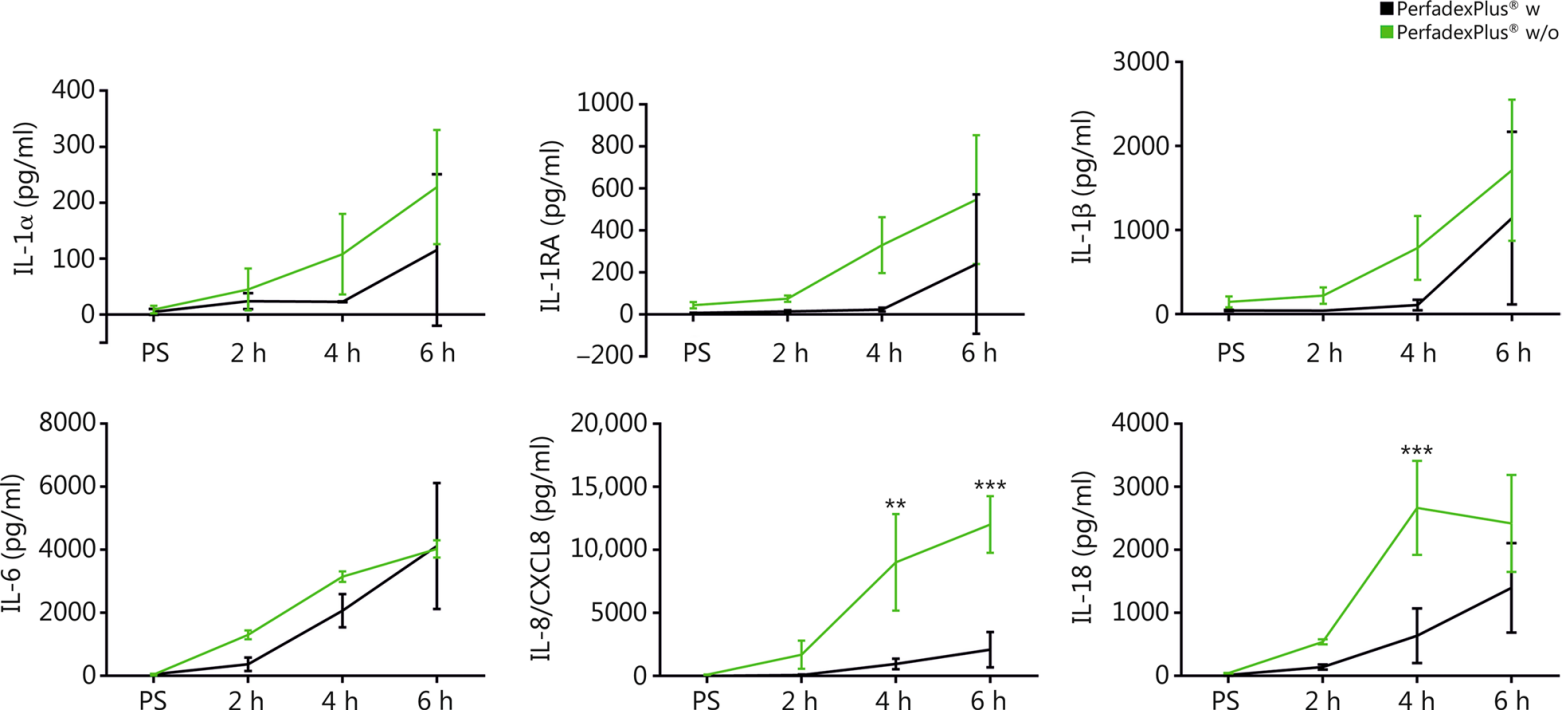
Relevant cytokines of ex vivo perfused limbs, including IL-1α, IL-1RA, IL-1β, IL-6, IL-8/CXCL8, and IL-18. For each figure, *n* = 3 was analyzed. Data of the perfusion group with (black) and without (green) medication were shown as mean values and standard deviations. Repeated measures two-way ANOVA and Tukey’s multiple comparison test were used to analyze grouped data over time. A multiple-adjusted *P*-value was given for each comparison. ^**^P < 0.01, ^***^P < 0.001. PS perfusion start, w with medication, w/o without medication

Cytokines also showed a time-dependent increase, which was also less pronounced in the w group, but only led to a significant difference in IL-8 for 4 h (*P* < 0.01) and 6 h (*P* < 0.001) and IL-18 for 4 h (*P* < 0.001) (Fig. 5). The levels of IL-1α [(115.7 ± 135.7) pg/ml (w) vs. (228.0 ± 102.1) pg/ml (w/o)], IL-1RA [(240.7 ± 332.0) pg/ml (w) vs. (548.0 ± 307.3) pg/ml (w/o)], IL-1β [(1144.0 ± 1029.0) pg/ml (w) vs. (1713.0 ± 841.5) pg/ml (w/o)], IL-6 [(4121.0 ± 2001.0) pg/ml (w) vs. (4027.0 ± 273.3) pg/ml (w/o)], IL-8 [(2095.0 ± 1411.0) pg/ml (w) vs. (12,020.0 ± 2263.0) pg/ml (w/o)] were highest at the end of perfusion both in the w group and w/o group, and IL-18 in the w group and w/o group reached the highest level at 6-hour and 4-hour, respectively [(1398.0 ± 711.2) pg/ml vs. (2668.0 ± 745.6) pg/ml] (Fig. 5). Results from further analyzed cytokines were depicted in Additional file 1: Table S2.

### Static storage

#### ***Temperature-variable, static storage***—***constant weight with resting cell activity***

Similar to the perfusion groups, initial surface temperatures were (22.0 ± 1.7) °C (CS) and (23.6 ± 1.7) °C (RT), with the RT group temperature dropping very slowly to (21.9 ± 0.4) °C by the end of the experiment. In contrast, CS limbs were cooled very quickly and reached (5.9 ± 3.1) °C after the first hour, which remained almost constantly throughout the entire experiment, also showing homogeneous temperature distribution (Additional file 1: Fig. S2a). Deep intramuscular temperature showed comparable decreases, starting at (29.6 ± 0.4) °C (CS) and (31.8 ± 1.2) °C (RT), and with corresponding final temperatures of (4.4 ± 2.4) °C (CS) and (23.6 ± 0.7) °C (RT) (Additional file 1: Fig. S2b). There were no significant differences between the groups in terms of BGA, serum markers, and cytokines, and their initial values did not change over the course of static storage, neither in relation to time nor temperature. In both groups, the initial pH of (6.8 ± 0) was at the end below the detection limit, pCO_2_ was almost constant at an average of (30.0 ± 3.7) mmHg (CS) and (23.0 ± 0.9) mmHg (RT). In contrast, pO_2_ increased from (90.0 ± 13.3) mmHg (CS) and (106.0 ± 26.7) mmHg (RT) to (162.7 ± 18.2) mmHg (CS) and (155.0 ± 14.7) mmHg (RT) (Additional file 1: Fig. S3a). Glucose, as well as sodium and potassium, showed no relevant changes during cold storage (Additional file 1: Fig. S3b); the same applied to cytokines (Additional file 1: Fig. S4 and Table S3). Initial lactate was (3.5 ± 0.3) mmol/L in the CS group and (3.7 ± 0) mmol/L in the RT group, and the CS group fell below the detection limit at the end (Additional file 1: Fig. S3c). Serum markers changed for LDH from (2505.0 ± 893.5) U/L to (2477.0 ± 274.7) U/L (RT) and (2424.0 ± 538.5) U/L to (3740.0 ± 1049.0) U/L (CS), for myoglobin from (365.3 ± 185.1) µg/L to (308.3 ± 139.7) µg/L (RT) and (304.7 ± 66.1) µg/L to (353.0 ± 145.3) µg/L (CS), and for CK from (13,012.0 ± 2833.0) U/L to (19,490.0 ± 4570.0) U/L (RT) and (18,554.0 ± 5204.0) U/L to (26,025.0 ± 9835.0) UL (CS) (Additional file 1: Fig. S5a).

There were also no relevant changes or differences between the groups with regard to COP, weight gain, and joint mobility. COP remained stable, as did initial weight, while joint mobility decreased from (47.3 ± 11.0) ° to (70.0 ± 17.3) ° (CS) and (54.7 ± 4.6) ° to (83.3 ± 40.4) ° (RT) for the knee and from (58.3 ± 22.6) ° to (84.7 ± 22.5) ° (CS) and (73.7 ± 17.0) ° to (88.3 ± 18.9) ° (RT) for the ankle, but these were not significant (Additional file 1: Fig. S5b). Histological evaluations showed a time-dependent increase in edema, similar to perfusion limbs. However, in contrast to the perfused limbs, which showed hardly any necrosis, there was a clear increase in necrosis, which was most pronounced in the RT group (Additional file 1: Table S1).

### EVEP may support peripheral nerve regeneration

**Fig. 6:**
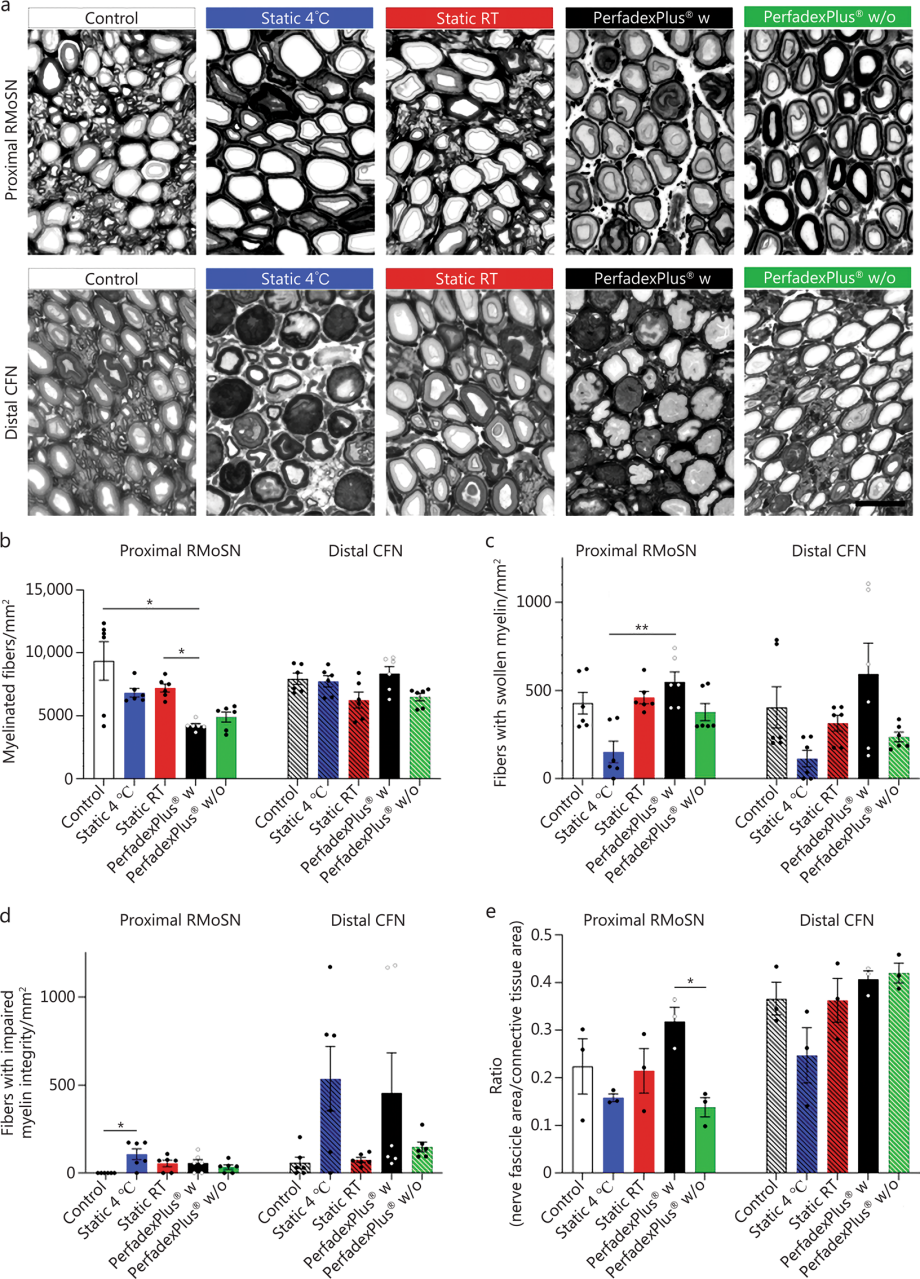
Stereological analysis of nerve segments of the proximal sciatic nerve muscle branch (unpatterned bars) and distal common fibular nerve (hatched bars). **a** Representative photomicrograph of toluidine blue-stained semi-thin cross sections of segments dissected from porcine proximal sciatic nerve muscle branch (RMoSN, top) and distal common fibular nerve (CFN, bottom). Scale bar = 10 µm. **b** Density of myelinated fibers (myelinated fibers/mm^2^). **c** Density of fibers showing a swollen myelin sheath. **d** Density of fibers with impaired integrity of the myelin sheath. **e** The area ratio between the nerve fascicles surrounded by perineurium and the epineurium, within the analyzed nerve segments. For Figs. **b–d**, data were subjected to a non-parametric Kruskal-Wallis test followed by Dunn’s multiple comparisons post-hoc test. The number of evaluated samples per condition was *n* = 6, harvested from *n* = 3 limbs per condition. For Fig. **e**, data were subjected to parametric one-way ANOVA followed by Tukey’s multiple comparisons post-hoc test. The number of evaluated sections per condition was *n* = 3. ^*^P < 0.05, ^**^P < 0.01. RT room temperature, w with medication, w/o without medication

The following data report values from the time at 6 h of storage or EVEP, respectively. On representative photomicrographs of cross-section details of the axonal myelin profiles, myelin profiles in the proximal RMoSN showed a rather homogeneous profile (Fig. 6a, upper row), while myelin profiles in the distal CFN showed visible irregularities (Fig. 6a, lower row). Such irregularities comprise an enlarged myelin layer in swollen myelin profiles, which were characterized by less intensive myelin staining, indicating less densely packed myelin proteins and impaired myelin integrity, with fibers demonstrating myelin constrictions within the axoplasm and a blurring of the lamellar sheath structure. Quantification of myelinated fibers with regular myelin profile/mm^2^ in the proximal RMoSN of the EVEP w group was significantly lower compared to the control and static RT groups, while distal nerve segments of the CFN showed no significant differences (Fig. 6b). Quantification of the density of myelinated fibers with swollen myelin sheaths showed the lowest values in the proximal and distal nerve samples when stored statically at 4 °C, resulting in a statistically significant difference compared to the EVEP w group in proximal nerve samples (Fig. 6c). Quantification of impaired myelin sheath fiber showed negligible values for the control, whereas proximal nerve samples were significantly increased when stored statically at 4 °C (Fig. 6d). In samples of the distal CFN, an increase in the respective fiber profiles was observed in the groups with static storage at 4 °C and EVEP w group, but this was not significantly increased, especially in comparison to the control (Fig. 6d). In the proximal RMoSN, EVEP w/o group showed the highest decrease in the ratio of nerve fascicle area to connective tissue area, indicating edema formation in the nerve tissue in comparison to control values; and this was also significantly lower compared to the EVEP w group, with values even higher than the control group (Fig. 6e). Distal nerve cross sections showed no significant differences between groups and had overall less edema than the proximal nerve cross sections (Fig. 6e).

**Fig. 7:**
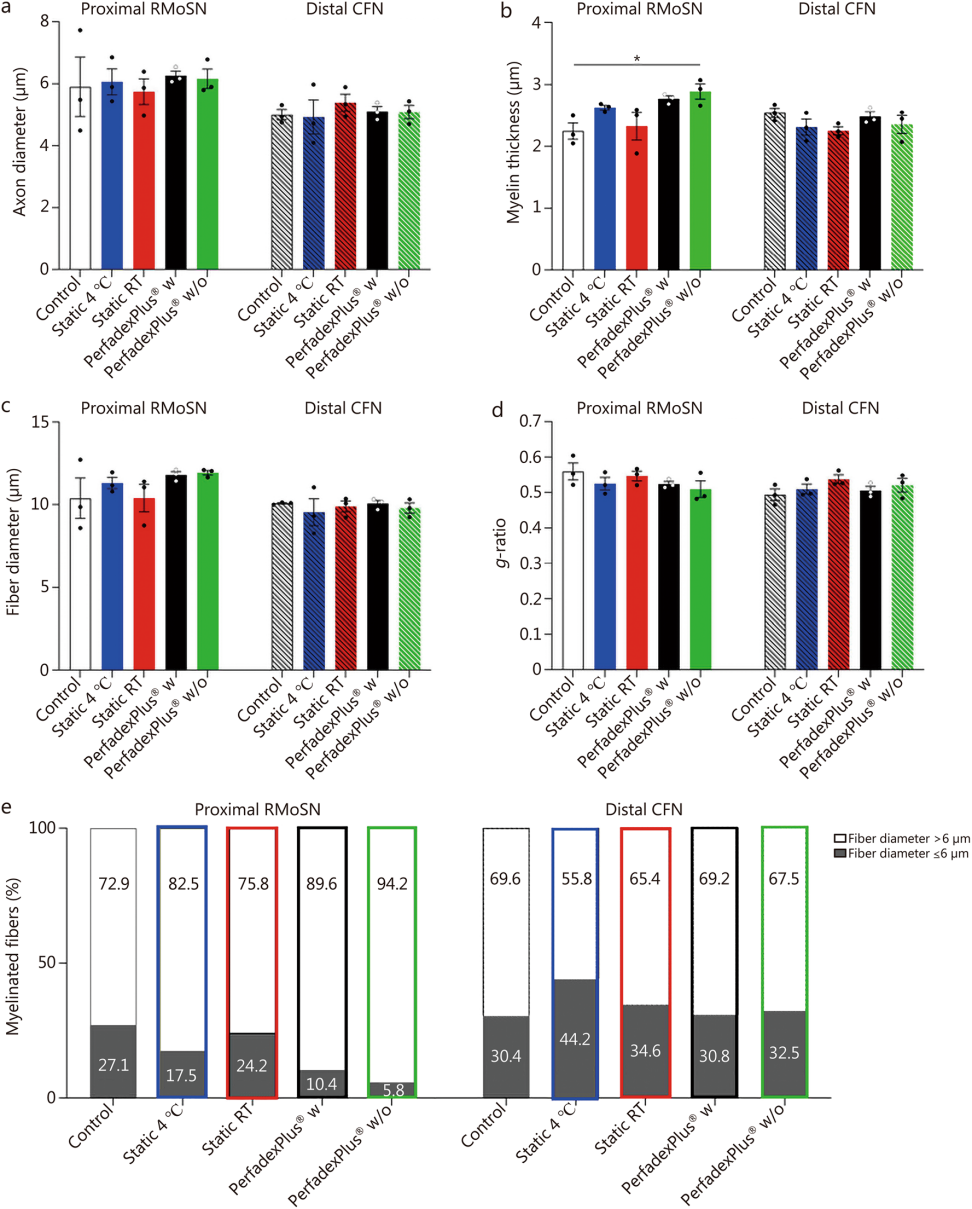
Morphometric analysis of nerve segments of the proximal sciatic nerve muscle branch (RMoSN, unpatterned bars) and distal common fibular nerve (CFN, hatched bars). **a** Axon diameter. **b** Myelin thickness. **c** Fiber diameter. **d** *g*-ratio. **e** The percentile distribution of nerve fiber diameters within the analyzed nerve segments dissected from the proximal RMoSN and the distal CFN. Stacked column graphs showed the percentage of nerve fibers with a diameter ≤ 6 µm (grey scales) and > 6 µm (black). Data was subjected to parametric one-way ANOVA followed by Tukey’s multiple comparisons post-hoc test. The number of evaluated samples per condition was *n* = 80 axons from *n* = 3 animals. ^*^P < 0.05. RT room temperature, w with medication, w/o without medication

After stereological evaluation, the morphometric properties of the axons were analyzed. Axon diameter did not differ significantly between the groups in the two nerves examined (Fig. 7a). However, myelin thickness was significantly increased in the proximal RMoSN in the EVEP w/o group compared to the control (Fig. 7b), but did not lead to significant differences in fiber diameter (Fig. 7c), which is derived from axon diameter plus myelin thickness. *G*-ratio, which is estimated by dividing the axon diameter by the diameter of the myelinated fiber, showed no significant differences between the groups (Fig. 7d). Percentile distribution of nerve fiber diameters in the different groups showed the highest number of nerve fibers with a diameter of more than 6 µm in proximal RMoSN in the EVEP w/o group, while the values in the EVEP w group were clearly (but not significantly) reduced in comparison (Fig. 7e). Percentile distribution of fibers in the distal CFN was more homogeneous between groups (Fig. 7e).

## Discussion

### Beyond transplantation: trauma-centered EVEP for military and civilian applications

In contrast to many other trials, this study investigates early pathophysiological changes following traumatic limb amputation in a porcine model, aiming to simulate and understand the implications of EVEP for trauma and military medicine, specifically targeting limb salvage and regenerative strategies, not transplantation [28]. To the best of our knowledge, this study constitutes one of the initial systematic investigations combining continuous normothermic perfusion of amputated limbs with comprehensive analysis comprising molecular, biochemical, and histological assessments over a defined time course. The study directly evaluates tissue viability, inflammatory responses, and early neural changes, including peripheral nerve histomorphometry and cytokine profiling, following traumatic limb amputation. The model is specifically designed to simulate both military and civilian trauma conditions. This trauma-focused perspective distinguishes the approach in this study from transplant-oriented perfusion protocols and is aligned with the hypothesis outlined in the previously published concept paper [29], which proposed EVEP as a bridge-to-decision or bridge-to-repair tool in the context of battlefield or civilian limb trauma. In addition, we assessed the impact of clinically relevant pharmacologic interventions (e.g., methylprednisolone, Iloprost) on tissue preservation, vascular resistance, and inflammation. Conducting human studies involving traumatic limb amputation in combination with extended ex vivo perfusion and pharmacologic interventions presents substantial ethical and logistical barriers. A preclinical porcine model offers a scientifically and ethically appropriate alternative to explore mechanistic processes and evaluate safety prior to translation into clinical settings. Given the close anatomical and physiological similarity between pigs and humans, the model provides clinically relevant data with high translational value. Additionally, the findings of this study provide initial evidence that specific cytokine profiles observed under EVEP may reflect the early onset of regenerative responses, such as those associated with WD, following traumatic peripheral nerve injury. To our knowledge, this represents a novel observation that has not been previously reported in the context of EVEP.

Overall, the controlled experimental evidence generated by this porcine model proof-of-concept study provides a robust foundation for translational research and establishes a scientifically based platform to investigate targeted therapeutic interventions, including clinically relevant pharmacological modulation and peripheral nerve tissue responses to significantly improve the outcome following traumatic limb loss, which will also benefit the field of transplantation.

### Basic considerations for EVEP

The current lack of replantation options in military operations with high amputation rates could be remedied by the development of a portable EVEP device. Although mechanisms of traumatic amputations in the military environment often differ drastically from those in the civilian population due to non-explosive mechanisms, the increasing number of harmful activities affecting both civilian and military populations, mass casualties, and military events worldwide clearly show that military medicine must also be transferred to the civilian population (such as the Boston-Marathon, the Ukraine war [1]. In order to enable the prompt realization of a clinically applicable EVEP, we have concentrated on translation-relevant aspects as part of the establishment process. Therefore, the porcine model was selected as it best reflects human anatomy in terms of size, tissue quality, and hemodynamics [30]. In addition to peripheral nerves that need to be prepared for optimal acceptance of freshly ingrowing axons towards regeneration, which is a key uniqueness of limb replantation, muscles are the most sensitive marker for determining the optimal perfusion protocol due to their short ischemia time [17]. In contrast to the literature, which primarily describes research on anterior extremities in the context of EVEP, we decided to use muscle-rich hind limbs, because if valid results are obtained, these can be easily transferred to the less muscle-rich upper extremities without further adjustments [19, 20, 31]. To realistically simulate traumatic limb amputation, the EVEP was preceded by a clinically relevant 2-h warm ischemia phase, reflecting documented prehospital delays of up to 2–4 h, which in the military trauma field result from emergency care of patients with severe combat injuries and their evacuation to a role 2 or 3 damage control station, and in the civilian field occur particularly in rural or resource-poor areas with limited emergency infrastructure and no air evacuation [29, 32, 33]. EVEP was performed using PerfadexPlus®, whose properties proved to be ideal by ensuring function and integrity of endothelium-rich organs, protecting the microvasculature from IRI and pathological leukocyte-endothelial interactions, and thus counteracting edema formation and thrombogenesis [31, 34–36]. To progressively identify the optimal perfusion protocol, the drugs known from solid organ transplantation were added to one part of the perfusion group and finally compared with the gold standard, static cold storage, and the worst-case scenario, static storage at RT [12, 13].

### Defined perfusion parameters

Perfusion parameters to be defined were based on physiological criteria, taking current literature into account, in particular pulsatile flow, which minimizes continuous flow-associated vascular dysfunction. Flow rates described varied between 5 and 500 ml/min regardless of the size or weight of the perfused limb [36]. In order to establish an individually customizable and therefore universally applicable protocol, EVEP was performed at 0.1 ml/(min·kg) limb weight, so that flows comparable to those generated by Werner et al. [21]. General perfusion pressures also varied between 30 and 120 mmHg, whereby perfusion pressures used in our protocol (w: 110 mmHg, w/o: 91 mmHg) were within the normal physiological range for lower in vivo extremity perfusion [7, 37, 38]. Using these pressure ranges, it was possible to avoid the extremes described in the literature, where both hypoperfusion (MAP < 70 mmHg) and hyperperfusion (MAP > 120 mmHg) impair the integrity of the microcirculation due to insufficient oxygen supply and endothelial cell damage caused by excessive shear stress, to ensure adequate tissue oxygenation and sufficient capillary flow to maintain tissue viability during ex-vivo perfusion [39]. As with solid organs, the optimal temperature for EVEP is controversial, ranging from 4 to 37 °C [17, 36]. Based on the physiological porcine temperature, the perfusate was adjusted to 38–39 ℃, as physiological cell metabolism and limb functionality can only be guaranteed under normothermic conditions [30]. These are also basic prerequisites to fully utilize the extended diagnostic-therapeutic possibilities of EVEP in the future, such as reassessment for replantation, for example, after radical wound debridement of an explosive amputation, which is often associated with considerable soft tissue trauma, tissue loss, and extensive contamination [17, 29]. For this, hypothermic perfusion is unsuitable as it simulates pseudoresistant hemorrhage due to vasoconstriction and makes adequate wound care impossible. By implementing thermography in EVEP, we established a non-invasive, innovative, and highly relevant method that can be used in a variety of ways. Temperature losses due to technical factors can be identified and thus minimized (such as optimal tube lengths), and continuous surface temperature analysis compared to the deep temperature enables the localization of pathophysiological non-/hypo-perfused areas, which can also be used to assess replantability [36, 37]. In this context, it is important to note that a surface-to-core temperature difference of approximately 10 ℃, as also observed in this study, is well-documented in the literature, considered physiologically normal, and does not in itself indicate insufficient perfusion [40]. Rather, it is a progressive or abrupt decline in temperature during perfusion that may suggest compromised flow or metabolic failure. In this experiment, no such pathological temperature shifts were detected over time, supporting the interpretation that tissue perfusion remained stable throughout the entire perfusion period.

### Perfusate components and supportive additives provide a physiological environment

Perfusate components maintained the ex vivo environment within a physiological range, which is particularly essential for valid functionality assessment, such as muscle contraction and nerve tissue condition [12, 13, 41]. Contrary to the literature, which describes 100% oxygen application with pO_2_ of > 500 mmHg [28, 36], target values of 150–250 mmHg were aimed based on experiences with extracorporeal circulation [42], at which metabolic activities with CO_2_ production are ensured and neither lead to cellular oxygen deficiency nor to cytotoxic reactions of reactive oxygen species (ROS). Additionally, a multivitamin complex as ROS scavenger was applied to counteract potentially cell-damaging effects, inflammatory responses, and apoptosis [43], although this remains to be quantified in the future. Despite increasing lactate levels, pH was maintained physiologically by adding TRIS without causing toxic hypernatremia [44, 45]. Detectable metabolic activity and homogeneous surface temperature ruled out both severe hypoxia and hypermetabolism, as well as insufficient limb perfusion as a cause of rising lactate levels, which probably resulted from the two hours of warm ischemia and may accumulate in the ex vivo circulation due to missing renal or hepatic elimination, causing lactic acidosis in the absence of counter regulation [28, 45]. These different mechanisms also lead to disagreement as to whether lactate should be recognized as a reliable predictive marker for cellular stress and tissue hypoxia and thus for transplant outcome after ex vivo perfusion, as is the case for heart but not lung transplantation [46, 47]. This discrepancy highlights the need for a more refined interpretation of lactate dynamics; however, its diagnostic utility remains limited in the absence of complementary parameters such as lactate clearance rates or mitochondrial function assays (e.g., high-resolution respirometry, ATP quantification, or membrane potential analysis), which are essential to distinguish reversible metabolic adaptation from irreversible cellular injury. Clear differences in lactate and potassium accumulation between the two EVEP groups can be explained by mutual influences of potassium, glucose, and insulin. Potassium is involved in a variety of physiological processes relevant to EVEP, such as muscle function and neural signaling [48, 49], and is only released during traumatic or ischemic-apoptotic processes and could therefore serve as a possible surrogate parameter for cell damage in EVEP in the future [48, 49]. Glucose is used for cellular energy production via aerobic glycolysis if sufficient glucose and oxygen are present; in contrast, if these are lacking, anaerobic glycolysis leads to an increase in lactate. This emphasizes the importance of physiological potassium and glucose levels during EVEP, which should also be ensured with medication if required [50, 51]. However, glucose administration alone leads to hyperkaliemia due to osmotic effects, while combined with insulin, it lowers intravascular potassium level and intracellular glucose level [50, 51]. This is reflected in significantly lower GUR of the w group, whose metabolic activity and potassium levels were physiologically maintained by combined glucose/insulin administration, and consequently led to significantly delayed lactate increase. In comparison, lack of insulin administration in the w/o group resulted in intracellular glucose and energy deficiency, leading to significantly increased GUR with rapidly rising lactate levels as part of anaerobic glycolysis.

### Protective function of medication supplements

LDH, myoglobin, and CK are used as markers of muscle injury and accumulate in the same way as lactate due to non-elimination. After a 2-h ischemia period, the static storage and EVEP limbs showed comparable baseline values caused by muscle injuries during surgical amputation [28]. These muscle ends continued to release these markers into the perfusate, but their levels were significantly higher in the w/o group. Together with the rapid lactate increase and the significantly reduced metabolic activity, this can be interpreted as further evidence of increasing intracellular ischemia, to which cells of the w group were not exposed, which additionally received cell-protective methylprednisolone and multivitamins, so that LDH and CK remained almost constant. In addition, the w group presented significantly lower weight gain, which presumably led to a less pronounced compartment syndrome with associated tissue ischemia, also seen by constant potassium levels [52]. In future studies, a causal distinction between marker release from injured muscle ends or a compartment syndrome should be investigated, as this has therapeutic consequences (e.g., fasciotomy).

### A two-edged sword: pro-inflammatory cytokines as key players in IRI and edema development

In principle, cytokine release is subject to a multifactorial process, but in the context of ex vivo perfusion, it is particularly associated with IRI and hypoxia. As expected, both EVEP groups showed time-dependent increases in cytokines, which in the w group were mainly IRI-associated and presented clear, partly significantly lower levels, especially of the proinflammatory IL-1α, IL1-β, IL-6, IL-8, and IL-18, as well as the anti-inflammatory natural inhibitor IL-1RA, and could be attributed to the cell-protective effects of the drugs, especially methylprednisolone. In addition to the lack of protective medication in the w/o group, an additional hypoxia-induced increase appears to be responsible for significantly higher cytokine levels, as both lack of prostavasin administration and weight-associated compartment syndrome significantly minimize microcirculation [53, 54]. Based on clinical data from ex vivo organ transplantation, IL-8 can be used as a predictive outcome marker [55]. Further, the use of anti-inflammatory drugs [56] or of certain adsorbers that could have a positive effect on organ quality [57] is discussed. These approaches should be investigated in the future, with a view to further protocol optimization in EVEP [17].

With regard to edema development, both perfusion groups demonstrated clear weight increase, although this was only significant in the w/o group. According to current literature, perfusion pressure, COP, and temperature have a significant influence [36], but due to comparable data of the 2 perfusion groups, these aspects seem to recede into the background. This is in line with current literature, as weight gains of > 40% are also reported under lower flow and pressure conditions (e.g., < 30 mmHg) and hypothermic storage compared to our protocol [35, 58]. The setup in this study can also exclude venous congestion as a potential cause for edema development, which is prevented by passive suction with venous negative pressure due to the position gradient between the limb and reservoir. In addition to the application of cytoprotective and anti-inflammatory drugs, the cytokine release was a major difference and thus possibly the main cause of edema formation. This was significantly increased for IL-8 in the w/o group, which reduces cell integrity and thus simultaneously increases permeability [55]. Therefore, IRI and the associated immune response and cytokine release appear to be the most important determinants for the development and extent of weight gain. However, whether this is clinically relevant for EVEP appears questionable, given the current literature. Neither in cardiac nor in renal ex vivo perfusion is this a parameter used to assess organ transplantability [59]. Kidneys, for example, have been successfully transplanted even with a weight gain of > 30% [60]. However, this does not apply to lungs, where the extent of weight gain determines transplantability [61].

Another diagnostic tool of EVEP is joint mobility, which is clearly restricted in the w/o group. This may not only be due to mechanical blockage caused by the more pronounced edema, but also to the accumulation of acidic metabolic products (e.g., lactate) and the lack of intracellular ATP/glucose metabolism with irreversible linking of actin and myosin filaments in skeletal muscle cells, which corresponds to the same mechanism as rigor mortis [62].

### A two-edged sword: pro-inflammatory cytokines indispensable for WD to induce nerve regeneration

Limb amputation inevitably results in the complete transection of peripheral nerves that provide sensor-motoric abilities to the limb. This disrupts the supply of the distal axon by the perikaryon, leading to axonal collapse. Functional recovery now depends on the successful creation of a pro-regenerative milieu in the distal stump, which allows regenerating axons to enter the stump and to be directed to the denervated target tissues. This is enabled by highly coordinated cellular and molecular mechanisms known as WD [59]. Their first hours are characterized by the induction of this pro-regenerative environment, in which resident repair Schwann cells and fibroblasts secrete pro-inflammatory cytokines such as IL-1α, IL-1β, IL-6, IL-8, IL-18, and MCP-1 [24, 63, 64], which play an important role in recruitment and differentiation of leukocytes and macrophages [22, 24, 64, 65]. Working together, they are responsible for axonal swelling, dissolution of axonal membranes, removal of axonal and myelin debris, and subsequent formation of so-called bands of Bünger for axonal guidance, which is crucial for both successful functionality and avoidance of neuroma formation with subsequent development of phantom pain [63].

To successfully establish an optimal EVEP, it is fundamental to understand whether and how normothermic EVEP affects peripheral nerves and their WD compared to the gold standard, static cold storage. Therefore, we analyzed its effects on cytokine composition and morphology of peripheral nerve fibers for the first time, since changes in these areas can be the first steps towards the pro-regenerative environment for WD. EVEP groups showed markedly axonal swelling and lower fiber density compared to static-stored samples. However, further analyses are needed to clarify whether this is due to edema or can be considered as an early sign of neural remodeling. The observed morphological changes may be compatible with early features of WD and provide a valuable basis for future targeted studies to further explore this hypothesis. Furthermore, perfusion groups showed the pro-regenerative cytokine profile typical of WD, which is not observed in the static groups, especially when stored at 4 °C due to a lack of cell activity. Consistent with the literature, the w group showed a significant reduction in WD-associated pro-regenerative cytokine profile due to anti-inflammatory effects of methylprednisolone, as well as significantly less connective tissue edema compared to the w/o group [63].

## Conclusions

In summary, EVEP offers the well-recognized advantages of ex-vivo perfusion, in particular the significant reduction of cold ischemia time and associated IRI [63], but also the possibility of surgical debridement and the immediate initiation of antibiotic and antifungal therapy, in parallel with improved patient care, to the most important aspect, the change from “saving lives before limbs” to “save lives and limbs”. At the same time, EVEP creates microenvironmental conditions that may allow for ex vivo induction of WD by establishing a pro-regenerative environment, which is further enhanced by the omission of methylprednisolone, which is otherwise routinely applied during ex vivo solid organ perfusion to deliberately weaken the pro-inflammatory immune response. Overall, EVEP offers the tremendous opportunity not only to significantly increase the number of limbs replanted after amputations but also to significantly improve their success rate in terms of functional recovery and avoidance of neuropathic pain [23]. Successful outcomes could be further optimized by future identification of predictive (bio-)markers and implementation of individually tailored supportive strategies (e.g., WD-focused ex vivo pharmaceutical or cell therapy) [23, 63, 64].

This proof-of-concept study yielded significant and replicable results, paving the way for the identification of suitable analytical methods and the generation of high-quality data, underscoring the viability and reproducibility of the EVEP system. Future studies should include larger cohorts to enhance statistical power, facilitate formal power analyses for designing subsequent trials, and address more specific research questions to confirm transferability.

## Supplementary Information

**Additional file 1. Fig. S1** Representative histopathological images of edema formation and necrosis. **Fig. S2** Temperature measurements of statically stored limbs. **Fig. S3** Blood gas analysis of statically stored limbs. **Fig. S4** Relevant cytokines of statically stored limbs. **Fig. S5** Serum markers and edema relevant aspects of statically stored limbs. **Table S1** Histopathological scoring of edema formation and necrosis (mean ± SD). **Table S2** Additional cytokines of perfused limbs (pg/ml, mean ± SD). **Table S3** Additional cytokines of statically stored limbs (pg/ml, mean ± SD).

## Data Availability

All data generated or analyzed during this study are included in this published article and its supplementary information file. Further inquiries are available from the corresponding author upon reasonable request.

## Abbreviations

BGA: Blood gas analysis
CO_2_: Carbon dioxide
CK: Creatine kinase
CNF: Common fibular nerve
COP: Colloidal oncotic pressure
CS: 4 °C static cold storage
EVEP: Ex vivo limb perfusion
GUR: Glucose uptake rate
IRI: Ischemia reperfusion injury
LDH: Lactate dehydrogenase
MAP: Mean arterial pressure
MVP: Mean venous pressure
NaCl: Sodium chloride
O_2_: Oxygen
PS: Perfusion start
RT: Room temperature
RMoSN: Sciatic nerve muscle branch
SD: Standard deviation
SEM: Standard error of mean
SP: System pressure
SSS: Start static storage
ST: System temperature
TRIS: Tris(hydroxymethyl)aminomethane
VR: Vascular resistance
WD: Wallerian degeneration
