# Supplementary material for: Ex-vivo limb perfusion in military and civilian medicine: inspired by ex-vivo organ perfusion, pioneered for traumatic limb amputation and peripheral nerve regeneration
Source: Mil Med Res. 2025 Oct 29;12:72. doi: 10.1186/s40779-025-00656-6 (PMC12570661; doi:10.1186/s40779-025-00656-6)
Supplement: Supplementary file 1 — Additional file 1. Fig. S1 Representative histopathological images of edema formation and necrosis. Fig. S2 Temperature measurements of statically stored limbs. Fig. S3 Blood gas analysis of statically stored limbs. Fig. S4 Relevant cytokines of statically stored limbs. Fig. S5 Serum markers and edema relevant aspects of statically stored limbs. Table S1 Histopathological scoring of edema formation and necrosis (mean ± SD). Table S2 Additional cytokines of perfused limbs (pg/ml, mean ± SD). Table S3 Additional cytokines of statically stored limbs (pg/ml, mean ± SD). [file 40779_2025_656_MOESM1_ESM.pdf]

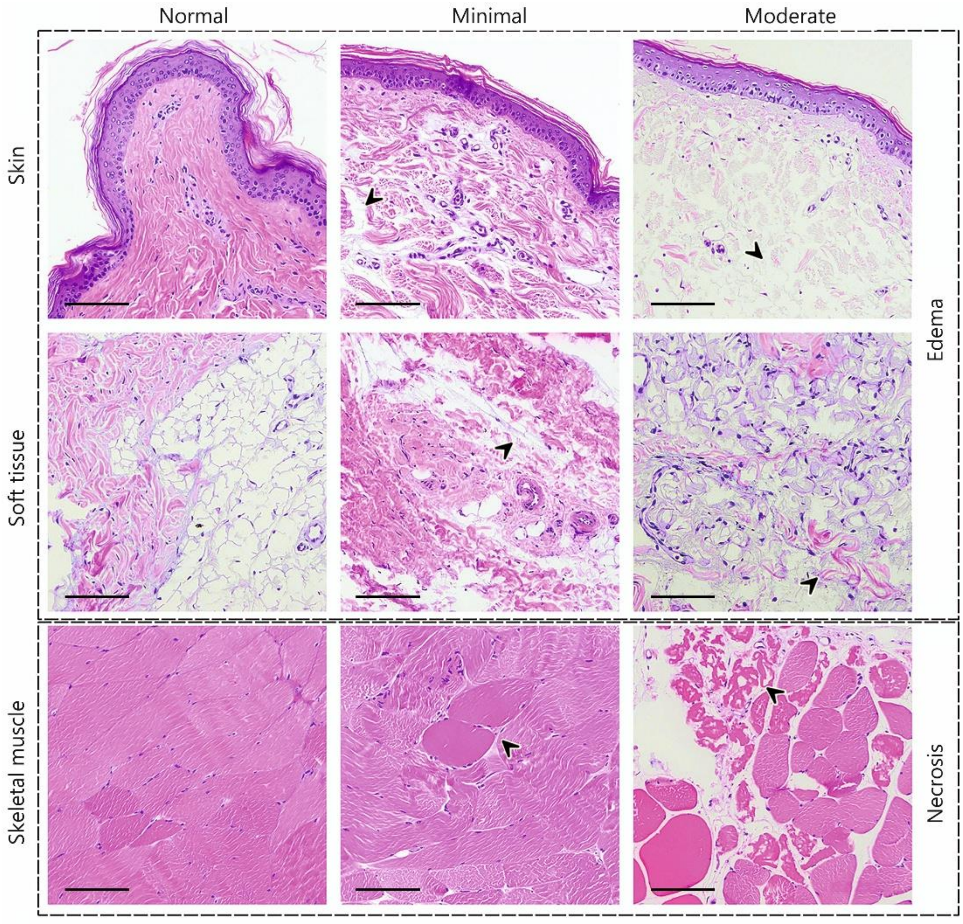

Fig. S1 Representative histopathological images of edema formation and necrosis. Representative images on histological changes of skin (first row), soft tissue (middle row), and skeletal muscle (lower row) according to the semi-quantitative grading (normal = left column, minimal changes = middle column, and moderate changes = right column). Arrows depicted the interstitial edema for skin and soft tissue panels and skeletal muscle necrosis in the skeletal muscle panels. Scale bar = 50 μm

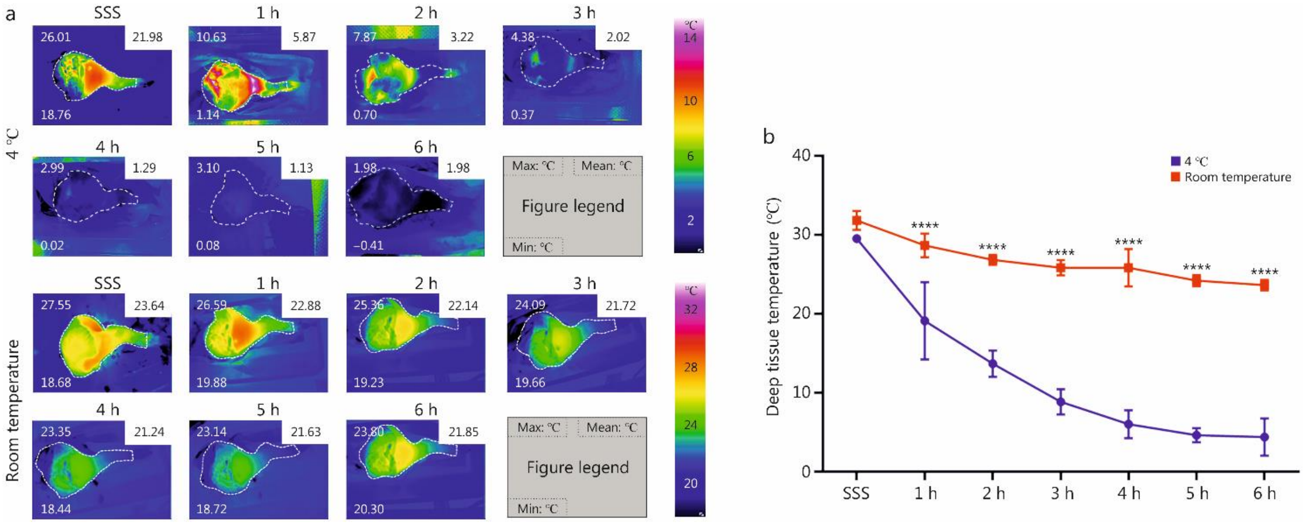

Fig. S2 Temperature measurements of static stored limbs**. a** Examples of the surface temperatures of statically stored limbs at room temperature and 4 °C. **b** The deep tissue temperature for statically stored limbs at room temperature (red) and 4 °C (blue). For each figure, n = 3 was analyzed. Data of the statically stored limbs at room temperature (red) and 4 °C (blue) were shown as mean values and standard deviations. Repeated measures two-way ANOVA and Tukey's multiple comparison test were used to analyze grouped data over time. A multiple-adjusted P-value was given for each comparison. The mean, maximum, and minimum temperatures were shown for surface temperatures, with the corresponding temperature color palette for both from 32 to 20 °C (room temperature) and from 14 °C to 2 °C (4 °C). SSS start static storage. $^{****}P < 0.0001$

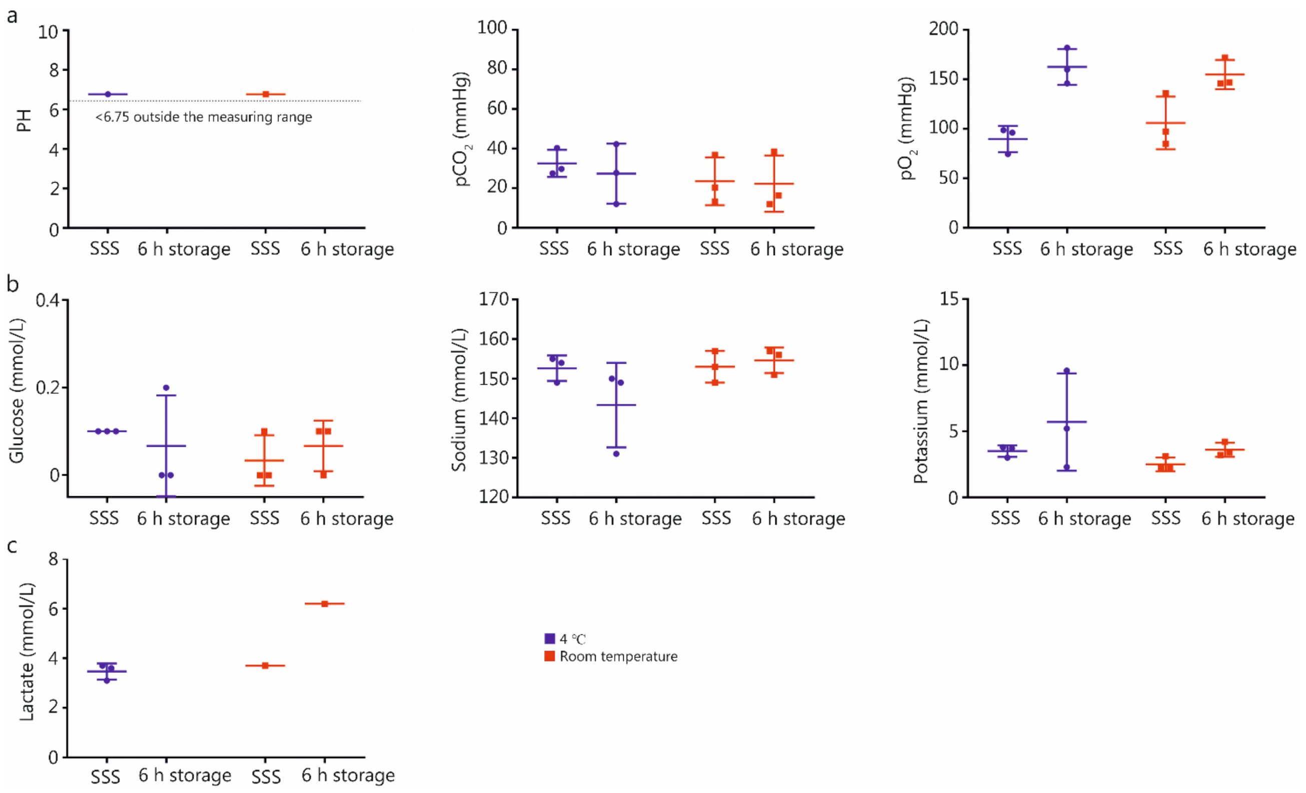

Fig. S3 Blood gas analysis of statically stored limbs. **a** pH, pCO_2, pO_2. **b** Glucose, Sodium, Potassium. **c** Lactate. For each figure, $n = 3$ was analyzed. pH and lactate were below the detection limit of < 0.1 during the experiment and could not be statistically evaluated. Data of the statically stored limbs at 4 °C (blue) and room temperature (red) were shown as mean values and standard deviations. Repeated measures two-way ANOVA and Tukey's multiple comparison test were used to analyze grouped data over time. A multiple-adjusted *P*-value was given for each comparison. CO_2 carbon dioxide, O_2 oxygen, SSS start static storage

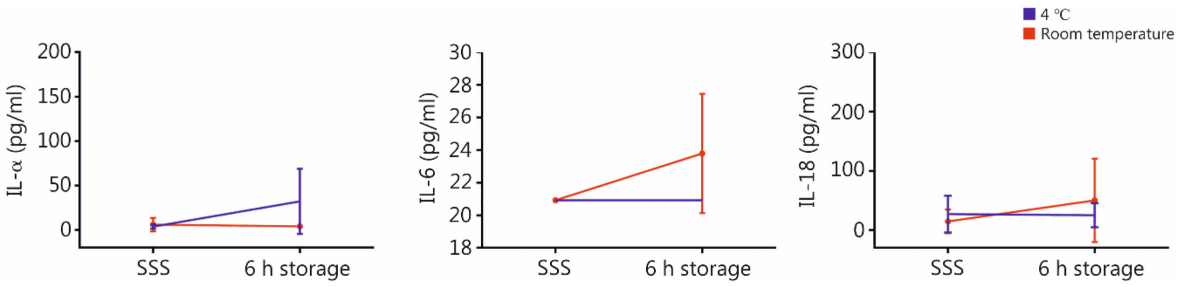

Fig. S4 Relevant cytokine of statically stored limbs. For each figure, $n = 3$ was analyzed. Data of the statically stored limbs at 4 ℃ (blue) and room temperature (red) were shown as mean values and standard deviations. Repeated measures two-way ANOVA and Tukey's multiple comparison test were used to analyze grouped data over time. A multiple-adjusted *P*-value was given for each comparison. SSS start static storage

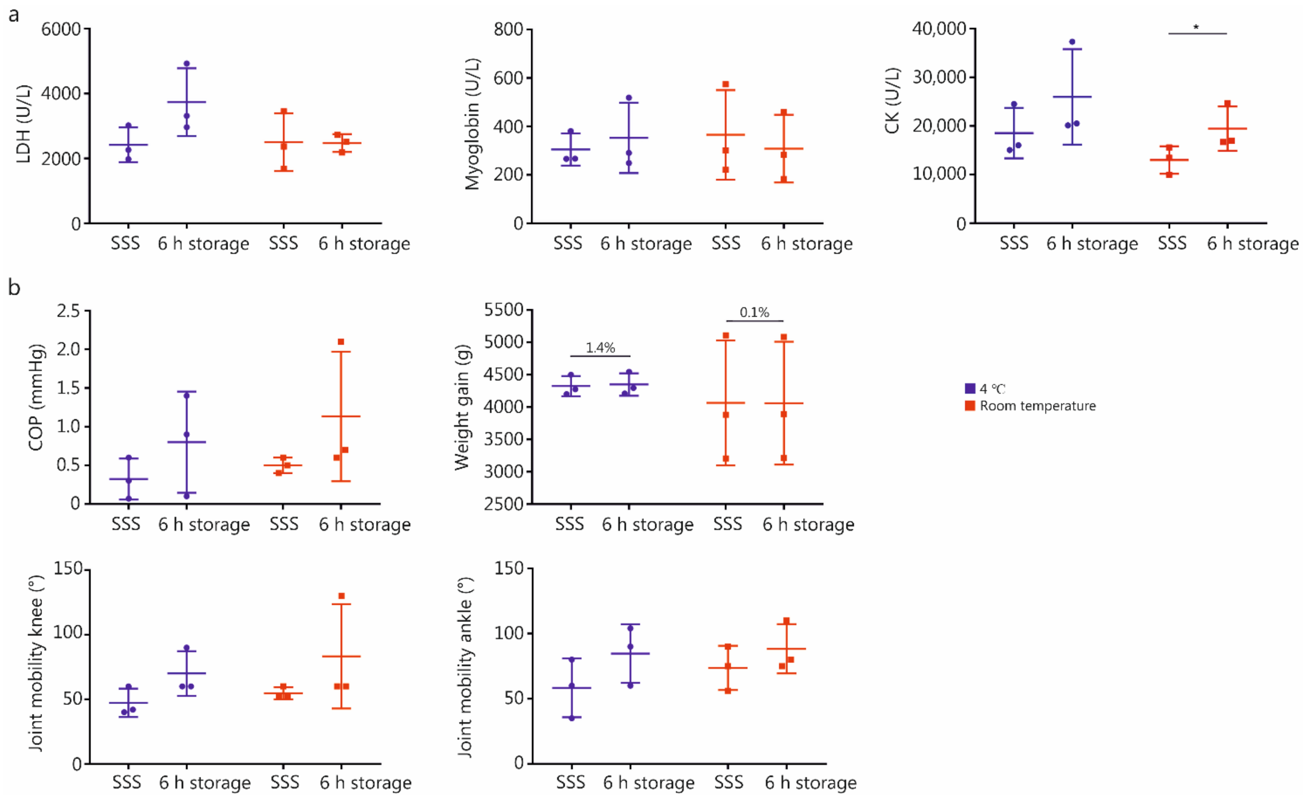

Fig. S5 Serum markers and edema relevant aspects of statically stored limbs. **a** Lactate dehydrogenase (LDH), Myoglobin, Creatine kinase (CK). **b** Colloid oncotic pressure (COP), Weight gain, Joint mobility of the knee, Joint mobility of the ankle. For each figure, $n = 3$ was analyzed. Data of the statically stored limbs at 4 ℃ (blue) and room temperature (red) were shown as mean values and standard deviations. Percentages stood for the weight gain between SSS and 6 h storage. Joint mobility data (knee and ankle) were analyzed for normal distribution and either subjected to a non-parametric Kruskal-Wallis test followed by Dunn's multiple comparisons post-hoc test or to parametric one-way ANOVA followed by Tukey's multiple comparisons post-hoc test. Repeated measures two-way ANOVA and Tukey's multiple comparison test were used to analyze grouped data over time. A multiple-adjusted *P*-value was given for each comparison. $^{*}P < 0.05$. SSS start static storage

Table S1 Histopathological scoring of edema formation and necrosis (mean ± SD)

Group	Edema			Necrosis		
	Control	2 h	6 h	Control	2 h	6 h
PerfadexPlus w/o	0.67 ± 0.58	0.66 ± 0.58	1.00 ± 0.00	0.00 ± 0.00	0.00 ± 0.00	0.34 ± 0.58
PerfadexPlus w	0.00 ± 0.00	0.00 ± 0.00	0.67 ± 1.16	0.00 ± 0.00	0.34 ± 0.58	0.00 ± 0.00
Static 4 °C	0.34 ± 0.58	0.34 ± 0.58	1.00 ± 1.00	0.00 ± 0.00	0.00 ± 0.00	0.34 ± 0.58
Static RT	0.00 ± 0.00	0.67 ± 1.16	0.67 ± 0.58	0.00 ± 0.00	0.00 ± 0.00	1.00 ± 1.00

The number of evaluated specimens per condition was $n = 3$. No statistically significant differences were visible via the Chi-square test. *RT* room temperature, *SD* standard deviation, *w* with medication, *w/o* without medication

Table S2 Additional cytokines of perfused limbs (pg/ml, mean ± SD)

Cytokines	Perfusion groups			
	PerfadexPlus w		PerfadexPlus w/o	
	PS	6 h	PS	6 h
GM-CSF	7.20 ± 0.00	7.20 ± 0.00	7.20 ± 0.00	7.20 ± 0.00
IFN-γ	3.19 ± 0.00	3.19 ± 0.00	3.19 ± 0.00	3.19 ± 0.00
IL-1α	4.83 ± 5.25	37.91 ± 22.70	8.98 ± 6.69	228.00 ± 102.10
IL-1β	43.19 ± 8.41	1144.00 ± 1029.00	145.50 ± 65.90	1713.00 ± 841.50
IL-1RA	7.71 ± 1.45	240.70 ± 332.00	44.18 ± 15.45	548.00 ± 307.30
IL-2	1.19 ± 0.00	1.19 ± 0.00	1.19 ± 0.00	1.19 ± 0.00
IL-4	0.04 ± 0.05	0.04 ± 0.05	0.51 ± 0.79	0.64 ± 0.94
IL-6	52.52 ± 6.28	4121.00 ± 2001.00	44.28 ± 25.06	4027.00 ± 273.30
IL-8/CXCL8	6.67 ± 0.00	2095.00 ± 1411.00	108.30 ± 16.41	12,020.00 ± 2263.00
IL-10	0.44 ± 0.17	9.62 ± 12.43	0.24 ± 0.00	56.76 ± 21.77
IL-12	13.29 ± 9.62	39.44 ± 22.90	26.47 ± 4.08	52.84 ± 19.93
IL-18	15.64 ± 16.00	1398.00 ± 711.20	46.14 ± 4.09	2423.00 ± 770.50
TNF-α	0.57 ± 0.00	0.57 ± 0.00	0.57 ± 0.00	0.57 ± 0.00

CXCL8 interleukin-8, *GM-CSF* Granulocyte-macrophage colony stimulating factor, *IFN-γ* interferon-γ, *IL* interleukin, *IL-1RA* interleukin-1 receptor antagonist, *PS* perfusion start, *SD* standard deviation, *TNF-α* tumor necrosis factor-α, *w* with medication, *w/o* without medication

Table S3 Additional cytokines of statically stored limbs (pg/ml, mean ± SD)

Cytokines	Static storage groups			
	Room temperature		4 °C	
	SSS	6 h	SSS	6 h
GM-CSF	7.20 ± 0.00	7.20 ± 0.00	7.20 ± 0.00	14.80 ± 10.70
IFN-γ	3.19 ± 0.00	229.30 ± 391.60	3.19 ± 0.00	279.60 ± 178.20
IL-1α	3.71 ± 2.37	32.10 ± 36.71	6.03 ± 7.53	4.14 ± 1.19
IL-1β	0.10 ± 0.00	0.10 ± 0.00	0.87 ± 1.10	0.10 ± 0.00
IL-1RA	1.43 ± 1.03	2.56 ± 0.46	2.43 ± 3.34	0.07 ± 0.00
IL-2	1.19 ± 0.00	1.28 ± 0.15	1.19 ± 0.00	1.19 ± 0.00
IL-4	0.01 ± 0.00	0.01 ± 0.00	0.01 ± 0.00	0.01 ± 0.00
IL-6	20.92 ± 0.00	20.92 ± 0.00	20.92 ± 0.00	23.80 ±3.66
IL-8/CXCL8	655.60 ± 0.00	655.60 ± 0.00	655.60 ± 0.00	655.60 ± 0.00
IL-10	0.24 ± 0.00	0.24 ± 0.00	0.24 ± 0.00	0.24 ± 0.00
IL-12	20.35 ± 5.72	3.37 ± 1.49	12.70 ± 1.33	3.30 ± 4.56
IL-18	26.94 ± 31.23	25.08 ±20.29	14.56 ± 20.16	50.08 ± 70.39
TNF-α	0.57 ± 0.00	1.87 ± 2.25	0.57 ± 0.00	19.90 ± 23.46

CXCL8 interleukin-8, *GM-CSF* Granulocyte-macrophage colony stimulating factor, *IFN-γ* interferon-γ, *IL* interleukin, *IL-1RA* interleukin-1 receptor antagonist, *SD* standard deviation, *SSS* start static storage, *TNF-α* tumor necrosis factor-α
